# Supplementary material for: Transplacental Transfer of Oxytocin and Its Impact on Neonatal Cord Blood and In Vitro Retinal Cell Activity
Source: Cells. 2024 Oct 19;13(20):1735. doi: 10.3390/cells13201735 (PMC11506339; doi:10.3390/cells13201735)
Supplement: Supplementary file 1 [file cells-13-01735-s001.zip › cells-3221876-supplementary.pdf]

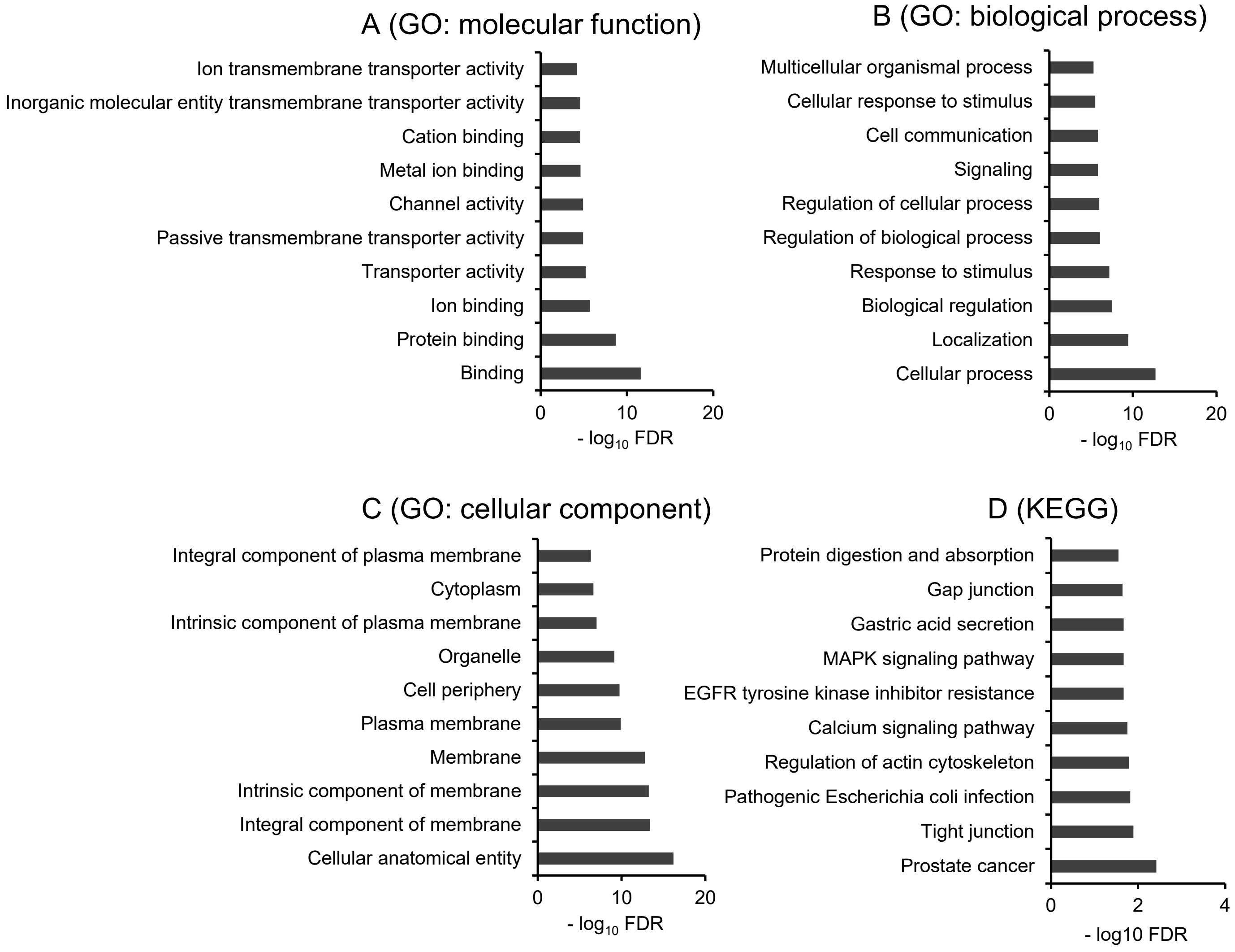

Supplementary Figure S1. Functional enrichment of down-regulated genes by oxytocin in human retinal pigment epithelial cells

Supplementary Table S1. Primers for Real-time PCR to validate upregulated genes

S. No.	Genes	Sequence 5'-3'
27	**IL7R FP**	CTCTGTCGCTCTGTTGGTCA
28	**IL7R RP**	CATCCACCCTATGAATCTGGC
29	**GABBR2 FP**	CAGATCCGCAACGAGTCAC
30	**GABBR2 RP**	GGATGTGACGGATGGACAGA
31	**ESM1 FP**	TTCGGGATGGATTGCAGAGA
32	**ESM1 RP**	CGGCAGCATTCTCTTTCACA
33	**CLEC18C FP**	GATCGGGCTCACCTACAAGA
34	**CLEC18C RP**	ATGTGCTCCTGGGCAAACT
35	**MMP1 FP**	ACAGCTTCCCAGCGACTCTA
36	**MMP1 RP**	AAACTGAGCCACATCAGGCA
37	**KISS1 FP**	CACAGGCCAGCAGCTAGAAT
38	**KISS1 RP**	TCCAGTTGTAGTTCGGCAGG
39	**XIRP1 FP**	CCATCCCCAGACCTTATCCC
40	**XIRP1 RP**	CCCACTTGGACCTTGTCTCT

Supplementary Table S2. Primers for Real-time PCR to validate downregulated genes

S. No.	Genes	Sequence 5'-3'
41	**FMO1 FP**	ATCTGCAAAAGCCAACCCTG
42	**FMO1 RP**	CTTGCAGTAGCACAAGCCAA
43	**SLC14A1 FP**	GCATCTGGGCTCTATGGCTA
44	**SLC14A1 RP**	CAACGCCATGTTGAAAGGGA
45	**ANPEP FP**	CGAAATGCCACACTGGTCAA
46	**ANPEP FP**	AGAACGAGCCACCACCATAA
47	**RNVU1-7 FP**	TTTTCCCAGGGCGAGGCTTA
48	**RNVU1-7 RP**	CCCCACTACCACAAATTATGCA

Supplementary Table S3. Primers for Real-time PCR to validate the genes involved in cholesterol biosynthesis

S. No.	Genes	Sequence 5'-3'
1	**ABCA4 FP**	AACCAGATCACCGCATTCCT
2	**ABCA4 RP**	GGCTGGTTTCAATGTCCCTTC
3	**RPE65 FP**	GCCGCTCACAGCTCATGTAA
4	**RPE65 RP**	TGGCTCAGATCCAACTTCAAAG
5	**CYP27A1 FP**	GGCAACGGAGCTTAGAGGAGAT
6	**CYP27A1 RP**	GCCTTGAACGAACAGCTGAAA
7	**CYP46A1 FP**	CCTTCTT CATTGCTGGTCACG
8	**CYP46A1 RP**	TCCATCACTGTGAACGCCAAG
9	**CYP11A1 FP**	GAACTTTTTGCCCCTGTTGGAT

10	**CYP11A1 RP**	TGTGCAGGACACTGACGAAGTC
11	**SREBP1 FP**	CGCAAGGCCATCGACTACATT
12	**SREBP1 RP**	TGCGCAGACTTAGGTTCTCCTG
13	**SREBP2 FP**	ATCGCTCCTCCATCAATGACA
14	**SREBP2 RP**	CTTGTGCATCTTGGCGTCTGT
15	**HMGCR FP**	GGTGTTCAAGGAGCATGCAAA
16	**HMGCR RP**	AGCCATTACGGTCCCACACA
17	**ABCA1 FP**	TCTTCCCACATTTTTGCCTGG
18	**ABCA1 RP**	CGATTCTCCCCAAACCTTTCC
19	**LDLR FP**	TGAAGTTGGCTGCGTTAATGTG
20	**LDLR RP**	ATTCGCCGCTGTGACACTTG
21	**SR-BI FP**	TCCA TCTACCCACCCAACGA
22	**SR-BI RP**	TGCAGGTGCTGACGTTCTGA
23	**SR-BII FP**	GGACTGCCTGCCTTTCGGTATA
24	**SR-BII RP**	CGGCATTGTCTGACGTATTGG
25	**APOE FP**	ACTGGGTCGCTTTTGGGATT
26	**APOE RP**	CAGTTGTTCCTCCAGTTCCG

Supplementary Table S4. Student's T-test p values for comparison between OXT treated and untreated samples and ANOVA p values for multigroup comparison

	**Student's T test *p* value**				
**Genes**	**10 uM OXT**	**1 uM OXT**	**0.1 uM OXT**	**0.01 uM OXT**	**ANOVA *p* value**
SRBI	0.086090287	0.21547073	0.201893551	0.065930317	0.02431
SRBII	0.013110752	0.006651134	0.061140075	0.004790122	0.00001
ABCA1	0.005236842	0.000173992	0.000276748	0.000721178	0.000001
ABCA4	0.000246148	0.003079386	0.000324804	0.116981847	0.00002
RPE65	0.032713819	0.002242301	0.001680135	0.001240909	0.000001
HMGCR	0.015038858	0.002982093	8.00607E-05	0.170898527	0.00278
SREBP1	0.084483758	0.001497761	2.735E-05	0.010011999	0.000001
SREBP2	0.030052405	0.031473767	0.000401631	0.018160958	0.00011
APOE	-	-	-	-	-
CYP11A1	0.059585408	0.008916604	0.005949754	0.007483146	0.00046
CYP27A1	0.058112006	0.029070943	0.000535448	0.002090097	0.000001
CYP46A1	0.051700383	0.119759424	0.019408109	0.248151064	0.04198
LDLR	0.059143406	0.131025083	0.174235095	0.05426916	0.0118
MMP-1	0.038362173	0.033696809	0.028909083	0.233273557	0.00008
GABBR2	0.20220795	0.047547349	0.012807853	0.02402434	0.0164
XIRP1	0.004275067	0.0089414	0.029904683	0.043287647	0.00088
KISS1	0.9116561	0.011267134	0.007152834	0.216212158	0.0279
RNVU1-7	0.057627367	0.03431215	0.002055194	0.003610348	0.0003
FMO1	0.000576905	0.00055443	0.001642324	0.00011001	0.000001

Supplementary Table S5. Up-regulated genes by oxytocin in HfRPE cells

Ensembl ID	Gene Symbol	log_2FC	FDR
ENSG00000235884	LINC00941	9.9874e-01	0.0085577
ENSG00000131016	AKAP12	9.9072e-01	4.7574E-10
ENSG00000261604	AC114947.2	9.8529e-01	0.00046132
ENSG00000144583	MARCH4	9.8285e-01	0.00021487
ENSG00000115221	ITGB6	9.7972e-01	0.00028073
ENSG00000175832	ETV4	9.7904e-01	0.0060646
ENSG00000170006	TMEM154	9.7645e-01	0.0088871
ENSG00000011347	SYT7	9.4652e-01	0.00016535
ENSG00000164761	TNFRSF11B	9.3943e-01	0.026331
ENSG00000163661	PTX3	9.3293e-01	1.3167E-07
ENSG00000132639	SNAP25	9.3207e-01	0.020979
ENSG00000154678	PDE1C	9.3115e-01	1.4579E-06
ENSG00000083454	P2RX5	9.2218e-01	0.034284
ENSG00000013619	MAMLD1	9.2131e-01	0.0016644
ENSG00000172995	ARPP21	9.0396e-01	0.0029819
ENSG00000186480	INSIG1	9.0344e-01	2.0107E-08
ENSG00000163814	CDCP1	8.9604e-01	0.00027441
ENSG00000120437	ACAT2	8.9117e-01	0.000015716
ENSG00000112972	HMGCS1	8.7442e-01	1.1636E-07
ENSG00000273003	ARL2-SNX15	8.6471e+00	0.0002681
ENSG00000235770	LINC00607	8.6234e-01	0.047836
ENSG00000170356	OR2A20P	8.6146e-01	0.041885
ENSG00000118523	CTGF	8.6138e-01	1.2935E-07
ENSG00000156453	PCDH1	8.5384e-01	0.00016025
ENSG00000146592	CREB5	8.5140e-01	0.00079619
ENSG00000079841	RIMS1	8.4668e-01	2.4127E-06
ENSG00000145777	TSLP	8.4435e-01	1.7158E-07
ENSG00000130164	LDLR	8.4252e-01	9.9415E-06
ENSG00000132196	HSD17B7	8.3677e-01	1.8703E-06
ENSG00000117525	F3	8.2777e-01	1.1636E-07
ENSG00000138131	LOXL4	8.1973e-01	0.0022693
ENSG00000052802	MSMO1	8.1591e-01	7.2748E-06
ENSG00000173166	RAPH1	8.0129e-01	1.3905E-06
ENSG00000185022	MAFF	7.9092e-01	0.00028073
ENSG00000138675	FGF5	7.9060e-01	1.1636E-07
ENSG00000134516	DOCK2	7.9023e-01	1.2257E-07
ENSG00000176697	BDNF	7.8490e-01	0.000014501
ENSG00000154127	UBASH3B	7.8304e-01	0.000009147
ENSG00000143882	ATP6V1C2	7.8115e-01	0.010691
ENSG00000276851	AC002401.4	7.5965e-01	0.042486
ENSG00000103489	XYLT1	7.5564e-01	0.0017505

ENSG00000113578	FGF1	7.5557e-01	1.1337E-06
ENSG00000127824	TUBA4A	7.5243e-01	0.000013058
ENSG00000166923	GREM1	7.5027e-01	5.0485E-06
ENSG00000115339	GALNT3	7.4989e-01	2.6357E-06
ENSG00000099194	SCD	7.4771e-01	0.000025046
ENSG00000273301	AC016717.2	7.4745e-01	0.00013312
ENSG00000185697	MYBL1	7.4244e-01	0.0038158
ENSG00000139278	GLIPR1	7.2974e-01	0.00037718
ENSG00000179546	HTR1D	7.2626e-01	0.0090134
ENSG00000166401	SERPINB8	7.1819e-01	0.0023816
ENSG00000180914	OXTR	7.1597e-01	0.00025137
ENSG00000052795	FNIP2	7.0345e-01	1.6316E-10
ENSG00000073008	PVR	7.0254e-01	0.000017323
ENSG00000011422	PLAUR	6.9753e-01	0.0017917
ENSG00000138623	SEMA7A	6.9516e-01	0.0038263
ENSG00000259207	ITGB3	6.9405e-01	0.026188
ENSG00000079459	FDFT1	6.9278e-01	2.0899E-06
ENSG00000164211	STARD4	6.9272e-01	0.00007148
ENSG00000067064	IDI1	6.9098e-01	0.000043641
ENSG00000178726	THBD	6.8303e-01	0.010133
ENSG00000148154	UGCG	6.8132e-01	0.00011878
ENSG00000197646	PDCD1LG2	6.7681e-01	0.010879
ENSG00000001630	CYP51A1	6.7115e-01	0.00019915
ENSG00000113361	CDH6	6.6963e-01	0.00028231
ENSG00000162772	ATF3	6.6728e-01	0.0037086
ENSG00000147155	EBP	6.6700e-01	5.0574E-06
ENSG00000104549	SQLE	6.6535e-01	6.2281E-06
ENSG00000150556	LYPD6B	6.6212e-01	0.0019245
ENSG00000106366	SERPINE1	6.6131e-01	0.03359
ENSG00000166073	GPR176	6.5765e-01	0.002558
ENSG00000157680	DGKI	6.5485e-01	0.00054169
ENSG00000133107	TRPC4	6.5194e-01	0.000047689
ENSG00000258757	AL133453.1	6.4960e-01	0.0054557
ENSG00000113389	NPR3	6.4681e-01	0.0001597
ENSG00000164741	DLC1	6.4614e-01	6.5209E-06
ENSG00000112414	ADGRG6	6.4320e-01	3.198E-08
ENSG00000113161	HMGCR	6.4188e-01	7.2444E-06
ENSG00000139211	AMIGO2	6.4073e-01	9.1401E-06
ENSG00000128606	LRRC17	6.3778e-01	0.000094192
ENSG00000187720	THSD4	6.3753e-01	0.0083723
ENSG00000182667	NTM	6.3404e-01	0.00017566
ENSG00000149218	ENDOD1	6.3329e-01	0.0014156
ENSG00000148082	SHC3	6.3277e-01	0.0010887

ENSG00000187123	LYPD6	6.3268e-01	0.0019363
ENSG00000135318	NT5E	6.2617e-01	0.022907
ENSG00000135074	ADAM19	6.2534e-01	0.00034974
ENSG00000128342	LIF	6.2320e-01	0.0094931
ENSG00000272414	FAM47E-STBD1	6.1607e-01	0.00039305
ENSG00000153976	HS3ST3A1	6.1241e-01	0.026188
ENSG00000067082	KLF6	6.1221e-01	0.0046697
ENSG00000166598	HSP90B1	6.1065e-01	0.0029358
ENSG00000096696	DSP	6.0948e-01	0.0010573
ENSG00000198108	CHSY3	6.0934e-01	0.026895
ENSG00000079931	MOXD1	6.0744e-01	5.0485E-06
ENSG00000184164	CRELD2	6.0545e-01	0.000094192
ENSG00000204516	MICB	6.0530e-01	0.026758
ENSG00000157193	LRP8	6.0137e-01	0.0077528
ENSG00000152495	CAMK4	6.0102e-01	0.0035023
ENSG00000152422	XRCC4	5.9997e-01	0.0070222
ENSG00000161791	FMNL3	5.9804e-01	0.0019363
ENSG00000044574	HSPA5	5.9592e-01	0.0045139
ENSG00000160752	FDPS	5.9524e-01	0.0021102
ENSG00000131473	ACLY	5.9318e-01	0.00014877
ENSG00000142871	CYR61	5.9192e-01	0.000088465
ENSG00000178922	HYI	5.8829e-01	0.022895
ENSG00000094804	CDC6	5.8647e-01	0.032069
ENSG00000136158	SPRY2	5.8634e-01	0.00065644
ENSG00000026508	CD44	5.8500e-01	0.0094931
ENSG00000168334	XIRP1	2.5915e+00	0.00015109
ENSG00000170498	KISS1	2.2393e+00	0.0011553
ENSG00000196611	MMP1	2.1173e+00	0.042855
ENSG00000168685	IL7R	1.6825e+00	8.2077E-11
ENSG00000157335	CLEC18C	1.6260e+00	0.018993
ENSG00000136928	GABBR2	1.5753e+00	0.000043641
ENSG00000164283	ESM1	1.5731e+00	0.00011844
ENSG00000148677	ANKRD1	1.4344e+00	1.1769E-09
ENSG00000183644	C11orf88	1.3815e+00	0.041232
ENSG00000203721	LINC00862	1.3754e+00	7.6586E-16
ENSG00000277247	AC083809.1	1.3567e+00	0.0016644
ENSG00000153233	PTPRR	1.3126e+00	0.00054169
ENSG00000198959	TGM2	1.2762e+00	1.2257E-07
ENSG00000144063	MALL	1.2370e+00	0.00000202
ENSG00000277954	AC092376.2	1.1967e+00	0.00016025
ENSG00000182261	NLRP10	1.1866e+00	0.01898
ENSG00000273221	AL355816.2	1.1816e+00	0.009184
ENSG00000171631	P2RY6	1.1743e+00	0.0013076

ENSG00000081181	ARG2	1.1610e+00	2.4127E-06
ENSG00000113070	HBEGF	1.1470e+00	8.1801E-11
ENSG00000100311	PDGFB	1.1378e+00	0.00019915
ENSG00000164171	ITGA2	1.1341e+00	2.3901E-10
ENSG00000282849	AL359834.1	1.1251e+00	0.015453
ENSG00000166292	TMEM100	1.1166e+00	0.017594
ENSG00000233117	LINC00702	1.1045e+00	6.3417E-07
ENSG00000110427	KIAA1549L	1.1036e+00	2.0693E-06
ENSG00000127329	PTPRB	1.0745e+00	2.535E-12
ENSG00000130477	UNC13A	1.0648e+00	0.00090217
ENSG00000170323	FABP4	1.0256e+00	0.049653
ENSG00000147573	TRIM55	1.0173e+00	5.8148E-06
ENSG00000125148	MT2A	1.0050e+00	0.00045592

log_2FC, log_2(fold change); FDR, false discovery rate that is a corrected p-value by the Benjamini-Hochberg procedure.

Supplementary Table S6. Down-regulated genes by oxytocin in HfRPE cells

Ensembl ID	Gene Symbol	log_2FC	FDR
ENSG00000170959	DCDC1	-0.58536	0.0094931
ENSG00000104081	BMF	-0.58937	0.032069
ENSG00000081803	CADPS2	-0.60147	0.030761
ENSG00000108924	HLF	-0.60503	0.0012464
ENSG00000110900	TSPAN11	-0.60611	0.0023816
ENSG00000188467	SLC24A5	-0.61796	0.0080078
ENSG00000136732	GYPC	-0.64646	0.043296
ENSG00000162989	KCNJ3	-0.64791	0.04329
ENSG00000187672	ERC2	-0.65153	0.011338
ENSG00000141401	IMPA2	-0.65429	0.033489
ENSG00000183785	TUBA8	-0.66125	0.00089273
ENSG00000269881	AC004754.1	-0.66568	0.040273
ENSG00000008277	ADAM22	-0.66839	0.012999
ENSG00000134765	DSC1	-0.67116	0.00033049
ENSG00000073282	TP63	-0.67705	0.0083723
ENSG00000180447	GAS1	-0.69446	0.00016025
ENSG00000066468	FGFR2	-0.70139	0.000014501
ENSG00000125872	LRRN4	-0.71109	0.040273
ENSG00000170962	PDGFD	-0.71569	6.7054E-06
ENSG00000114698	PLSCR4	-0.72874	0.0012101
ENSG00000100033	PRODH	-0.73521	0.026188
ENSG00000118322	ATP10B	-0.73955	0.0035734
ENSG00000130600	H19	-0.74064	1.8703E-06
ENSG00000166265	CYYR1	-0.74232	0.027541
ENSG00000169083	AR	-0.7485	0.039203

ENSG00000168453	HR	-0.77106	0.00044861
ENSG00000138080	EMILIN1	-0.80757	0.0026043
ENSG00000184408	KCND2	-0.81626	0.0023847
ENSG00000184809	B3GALT5-AS1	-0.82381	0.027178
ENSG00000180730	SHISA2	-0.83115	0.035136
ENSG00000205336	ADGRG1	-0.83663	0.049653
ENSG00000106333	PCOLCE	-0.84051	0.03096
ENSG00000171219	CDC42BPG	-0.84597	0.020581
ENSG00000279592	AC010653.2	-0.87347	0.040371
ENSG00000105409	ATP1A3	-0.877	0.0005431
ENSG00000156097	GPR61	-0.88474	0.036032
ENSG00000162267	ITIH3	-0.88815	0.0094715
ENSG00000174807	CD248	-0.92117	0.0030099
ENSG00000013297	CLDN11	-0.92291	0.0044293
ENSG00000145721	LIX1	-0.9469	1.1636E-07
ENSG00000182612	TSPAN10	-0.95958	0.000060343
ENSG00000253417	LINC02159	-0.96665	0.00011793
ENSG00000251491	OR7E28P	-1.0046	0.02013
ENSG00000166473	PKD1L2	-1.0268	0.0045139
ENSG00000198732	SMOC1	-1.0392	0.00023707
ENSG00000163331	DAPL1	-1.0674	2.7152E-09
ENSG00000147606	SLC26A7	-1.1516	1.8703E-06
ENSG00000124749	COL21A1	-1.1622	0.0054557
ENSG00000168874	ATOH8	-1.164	0.026188
ENSG00000198216	CACNA1E	-1.1999	0.032069
ENSG00000133392	MYH11	-1.2755	0.014687
ENSG00000126778	SIX1	-1.3333	0.0099313
ENSG00000010932	FMO1	-1.6798	0.00171
ENSG00000166825	ANPEP	-2.36	0.00072974
ENSG00000141469	SLC14A1	-2.362	0.00034974
ENSG00000206585	RNVU1-7	-4.1166	0.0088871

log_2FC, log_2(fold change); FDR, false discovery rate that is a corrected p-value by the Benjamini-Hochberg procedure. The minus sign with the fold change data denotes downregulation.

Supplementary Table S7. Enriched KEGG terms from up-regulated genes by OXT in HfRPE cells

**Term name**	**Term ID**	**P_{adj}**	**$-log_{10}$ (P_{adj})**
Metabolic pathways	KEGG:01100	5.493×10^{-11}	10.260208757933059
Steroid biosynthesis	KEGG:00100	2.965×10^{-10}	9.52802338202263
Terpenoid backbone biosynthesis	KEGG:00900	6.749×10^{-8}	7.1707924079289524
PI3K-Akt signaling pathway	KEGG:04151	4.012×10^{-7}	6.396613015701344
Calcium signaling pathway	KEGG:04020	4.500×10^{-5}	4.346772353328488
cAMP signaling pathway	KEGG:04024	2.673×10^{-4}	3.5730327987922217
Regulation of actin cytoskeleton	KEGG:04810	2.673×10^{-4}	3.5730327987922217
Estrogen signaling pathway	KEGG:04915	3.438×10^{-4}	3.4636350946754777

PPAR signaling pathway	KEGG:03320	4.908×10^{-4}	3.309058986245986
Arrhythmogenic right ventricular cardiomyopathy	KEGG:05412	4.908×10^{-4}	3.309058986245986
Synaptic vesicle cycle	KEGG:04721	4.908×10^{-4}	3.309058986245986
Complement and coagulation cascades	KEGG:04610	6.351×10^{-4}	3.197126706631979
ECM-receptor interaction	KEGG:04512	6.756×10^{-4}	3.1703110339129124
Hematopoietic cell lineage	KEGG:04640	8.855×10^{-4}	3.0527958208281993
Focal adhesion	KEGG:04510	1.169×10^{-3}	2.932283209403435
Proteoglycans in cancer	KEGG:05205	1.235×10^{-3}	2.9084565471529005
Neuroactive ligand-receptor interaction	KEGG:04080	1.407×10^{-3}	2.8515698299418273
Ras signaling pathway	KEGG:04014	1.924×10^{-3}	2.715845331753448
Pathways in cancer	KEGG:05200	1.997×10^{-3}	2.6995775865716922
Synthesis and degradation of ketone bodies	KEGG:00072	1.997×10^{-3}	2.6995775865716922
Fluid shear stress and atherosclerosis	KEGG:05418	2.372×10^{-3}	2.6247995971678524
Phagosome	KEGG:04145	3.041×10^{-3}	2.5170105348989837
GnRH secretion	KEGG:04929	3.266×10^{-3}	2.4859330642671384
JAK-STAT signaling pathway	KEGG:04630	3.931×10^{-3}	2.4054834929764155
Melanoma	KEGG:05218	4.100×10^{-3}	2.38716491246505
MAPK signaling pathway	KEGG:04010	4.100×10^{-3}	2.38716491246505
Thyroid hormone synthesis	KEGG:04918	4.301×10^{-3}	2.3664669300702172
Glioma	KEGG:05214	4.301×10^{-3}	2.3664669300702172
Alcoholism	KEGG:05034	5.465×10^{-3}	2.2624426785498937
Glycosaminoglycan biosynthesis - chondroitin sulfate / dermatan sulfate	KEGG:00532	5.465×10^{-3}	2.2624426785498937
Taste transduction	KEGG:04742	5.606×10^{-3}	2.2513692862277073
Human papillomavirus infection	KEGG:05165	5.643×10^{-3}	2.2485244171773244
Hypertrophic cardiomyopathy	KEGG:05410	6.223×10^{-3}	2.206011883646719
Dilated cardiomyopathy	KEGG:05414	6.866×10^{-3}	2.1632668677376152
Glycosaminoglycan biosynthesis - heparan sulfate / heparin	KEGG:00534	6.866×10^{-3}	2.1632668677376152
Rap1 signaling pathway	KEGG:04015	6.906×10^{-3}	2.1607755067291463
Prostate cancer	KEGG:05215	6.906×10^{-3}	2.1607755067291463
Aldosterone synthesis and secretion	KEGG:04925	6.930×10^{-3}	2.159283785119226
AGE-RAGE signaling pathway in diabetic complications	KEGG:04933	7.159×10^{-3}	2.145146187185454
Butanoate metabolism	KEGG:00650	8.121×10^{-3}	2.090383777319861
Glyoxylate and dicarboxylate metabolism	KEGG:00630	9.096×10^{-3}	2.041164854412673
AMPK signaling pathway	KEGG:04152	1.067×10^{-2}	1.9716326771139658
Neurotrophin signaling pathway	KEGG:04722	1.067×10^{-2}	1.9716326771139658
Osteoclast differentiation	KEGG:04380	1.199×10^{-2}	1.9211375490562366
Relaxin signaling pathway	KEGG:04926	1.254×10^{-2}	1.9017178921910014
Apelin signaling pathway	KEGG:04371	1.455×10^{-2}	1.8372810995351536
Bladder cancer	KEGG:05219	1.476×10^{-2}	1.8310457768869457
Gastric cancer	KEGG:05226	1.664×10^{-2}	1.7789473430994798
Breast cancer	KEGG:05224	1.664×10^{-2}	1.7789473430994798
Cytokine-cytokine receptor interaction	KEGG:04060	1.664×10^{-2}	1.7789473430994798
Phospholipase D signaling pathway	KEGG:04072	1.664×10^{-2}	1.7789473430994798
MicroRNAs in cancer	KEGG:05206	1.811×10^{-2}	1.7419994522905842
Huntington disease	KEGG:05016	1.811×10^{-2}	1.7419994522905842

Valine, leucine and isoleucine degradation	KEGG:00280	1.811×10^{-2}	1.7419994522905842
Hippo signaling pathway	KEGG:04390	1.811×10^{-2}	1.7419994522905842
Cocaine addiction	KEGG:05030	1.811×10^{-2}	1.7419994522905842
Ovarian steroidogenesis	KEGG:04913	1.870×10^{-2}	1.7280815004429235
Fatty acid metabolism	KEGG:01212	2.281×10^{-2}	1.6419042662967722
Cortisol synthesis and secretion	KEGG:04927	2.803×10^{-2}	1.552315442450255
Amphetamine addiction	KEGG:05031	3.185×10^{-2}	1.496916932204297
Epithelial cell signaling in Helicobacter pylori infection	KEGG:05120	3.221×10^{-2}	1.4920413759104951
EGFR tyrosine kinase inhibitor resistance	KEGG:01521	3.990×10^{-2}	1.399075703999735
ErbB signaling pathway	KEGG:04012	4.410×10^{-2}	1.3555307958725178
Insulin secretion	KEGG:04911	4.539×10^{-2}	1.343003874863852
Longevity regulating pathway	KEGG:04211	4.561×10^{-2}	1.3409329420229636
Gap junction	KEGG:04540	4.561×10^{-2}	1.3409329420229636
Morphine addiction	KEGG:05032	4.561×10^{-2}	1.3409329420229636
Rheumatoid arthritis	KEGG:05323	4.561×10^{-2}	1.3409329420229636
Bile secretion	KEGG:04976	4.591×10^{-2}	1.3380713568369411
TGF-beta signaling pathway	KEGG:04350	4.813×10^{-2}	1.3175405871726733
IL-17 signaling pathway	KEGG:04657	4.843×10^{-2}	1.3149235266239303
Endocrine resistance	KEGG:01522	4.968×10^{-2}	1.3038271261311332

KEGG, Kyoto Encyclopedia of Genes and Genomes database; P_{adj} or FDR (false discovery rate), a corrected p-value.

Supplementary Table S8. Enriched GO molecular function for up-regulated genes by OXT in HfRPE cells

**Term name**	**Term ID**	P_{adj}	$-\log_{10}(P_{adj})$
Binding	GO:0005488	3.626×10^{-41}	40.440518247687415
Protein binding	GO:0005515	3.289×10^{-33}	32.48291927768249
Ion binding	GO:0043167	1.445×10^{-13}	12.840083774670706
Cation binding	GO:0043169	4.711×10^{-11}	10.326890329108851
Signaling receptor binding	GO:0005102	8.569×10^{-11}	10.067069982211294
Metal ion binding	GO:0046872	8.860×10^{-11}	10.052586057685692
Signaling receptor activity	GO:0038023	2.449×10^{-10}	9.610979860507184
Molecular transducer activity	GO:0060089	2.449×10^{-10}	9.610979860507184
Catalytic activity	GO:0003824	7.523×10^{-10}	9.123601479679202
Cell adhesion molecule binding	GO:0050839	2.074×10^{-9}	8.683195852407941
Receptor regulator activity	GO:0030545	2.069×10^{-8}	7.684206759449206
Protein-containing complex binding	GO:0044877	4.420×10^{-8}	7.354624021820886
Growth factor receptor binding	GO:0070851	6.198×10^{-8}	7.207747275039933
Integrin binding	GO:0005178	7.594×10^{-8}	7.119531622247917
Transmembrane signaling receptor activity	GO:0004888	1.974×10^{-7}	6.704700081904414
Molecular function regulator	GO:0098772	6.315×10^{-7}	6.199600105304236
Receptor ligand activity	GO:0048018	6.632×10^{-7}	6.178362933480093
Signaling receptor activator activity	GO:0030546	7.101×10^{-7}	6.148672299588162
Growth factor activity	GO:0008083	2.852×10^{-6}	5.544902072205491
Intramolecular oxidoreductase activity	GO:0016860	3.669×10^{-6}	5.435419744142716
Insulin-like growth factor binding	GO:0005520	1.350×10^{-5}	4.869555505777967
Exogenous protein binding	GO:0140272	2.060×10^{-5}	4.68607660216971

Virus receptor activity	GO:0001618	2.060×10^{-5}	4.68607660216971
Calcium ion binding	GO:0005509	2.060×10^{-5}	4.68607660216971
Enzyme binding	GO:0019899	3.000×10^{-5}	4.522827401559904
Isomerase activity	GO:0016853	5.261×10^{-5}	4.278900087351218
Carbohydrate derivative binding	GO:0097367	1.024×10^{-4}	3.9896415865497645
Organic cyclic compound binding	GO:0097159	1.201×10^{-4}	3.920525482965963
Calmodulin binding	GO:0005516	1.348×10^{-4}	3.8703834532888903
Extracellular matrix binding	GO:0050840	1.348×10^{-4}	3.8703834532888903
Transition metal ion binding	GO:0046914	1.715×10^{-4}	3.7656889837818985
Growth factor binding	GO:0019838	2.602×10^{-4}	3.5847113908672483
Small molecule binding	GO:0036094	2.876×10^{-4}	3.541241900880014
Collagen binding	GO:0005518	2.876×10^{-4}	3.541241900880014
Identical protein binding	GO:0042802	3.608×10^{-4}	3.4427681780723143
Very-low-density lipoprotein particle receptor activity	GO:0030229	4.110×10^{-4}	3.3861530136859206
Heterocyclic compound binding	GO:1901363	4.838×10^{-4}	3.315298818111507
Oxidoreductase activity, acting on paired donors, with incorporation or reduction of molecular oxygen	GO:0016705	5.682×10^{-4}	3.2455325091436
Anion binding	GO:0043168	5.702×10^{-4}	3.24400608539834
Transferase activity, transferring acyl groups, acyl groups converted into alkyl on transfer	GO:0046912	6.172×10^{-4}	3.2095458355430253
Catalytic activity, acting on a protein	GO:0140096	1.250×10^{-3}	2.9030161104831858
Monooxygenase activity	GO:0004497	1.259×10^{-3}	2.8999731520120386
Nucleotide binding	GO:0000166	1.722×10^{-3}	2.763914284782142
Nucleoside phosphate binding	GO:1901265	1.722×10^{-3}	2.763914284782142
Vascular endothelial growth factor receptor 2 binding	GO:0043184	1.969×10^{-3}	2.705853890866936
Oxidoreductase activity, acting on paired donors, with incorporation or reduction of molecular oxygen, NAD(P)H as one donor, and incorporation of one atom of oxygen	GO:0016709	1.974×10^{-3}	2.7047132697360006
Glycosaminoglycan binding	GO:0005539	2.378×10^{-3}	2.6237687082737255
Protease binding	GO:0002020	3.123×10^{-3}	2.505469850559008
Low-density lipoprotein particle receptor activity	GO:0005041	3.679×10^{-3}	2.4342145271774376
Transferase activity	GO:0016740	3.679×10^{-3}	2.4342145271774376
Sulfur compound binding	GO:1901681	3.679×10^{-3}	2.4342145271774376
UDP-glycosyltransferase activity	GO:0008194	3.679×10^{-3}	2.4342145271774376
Neurotransmitter receptor regulator activity	GO:0099602	3.925×10^{-3}	2.4061652807426404
Vascular endothelial growth factor receptor binding	GO:0005172	3.925×10^{-3}	2.4061652807426404
Manganese ion binding	GO:0030145	3.925×10^{-3}	2.4061652807426404
Oxidoreductase activity	GO:0016491	3.925×10^{-3}	2.4061652807426404
Acetylcholine receptor regulator activity	GO:0030548	3.925×10^{-3}	2.4061652807426404
Cytokine receptor binding	GO:0005126	3.945×10^{-3}	2.4039770996612853
Platelet-derived growth factor receptor binding	GO:0005161	4.362×10^{-3}	2.3603118555296807

Enzyme regulator activity	GO:0030234	4.729×10^{-3}	2.325187205921824
Lipoprotein particle receptor activity	GO:0030228	4.741×10^{-3}	2.3241673528864473
Intramolecular oxidoreductase activity, transposing C=C bonds	GO:0016863	4.741×10^{-3}	2.3241673528864473
Heparin binding	GO:0008201	4.882×10^{-3}	2.31140208016566
Transmembrane receptor protein tyrosine phosphatase activity	GO:0005001	5.121×10^{-3}	2.2906711040499843
Transmembrane receptor protein phosphatase activity	GO:0019198	5.121×10^{-3}	2.2906711040499843
Cargo receptor activity	GO:0038024	5.576×10^{-3}	2.25366164251801
Prenyltransferase activity	GO:0004659	5.583×10^{-3}	2.253141468253114
Syntaxin binding	GO:0019905	5.622×10^{-3}	2.2501338663515127
Protein disulfide isomerase activity	GO:0003756	5.967×10^{-3}	2.224244016220836
Intramolecular oxidoreductase activity, transposing S-S bonds	GO:0016864	5.967×10^{-3}	2.224244016220836
Hydrolase activity	GO:0016787	6.620×10^{-3}	2.1791431595203967
Purinergic nucleotide receptor activity	GO:0001614	7.010×10^{-3}	2.1543094318997986
Nucleotide receptor activity	GO:0016502	7.010×10^{-3}	2.1543094318997986
Cytokine receptor activity	GO:0004896	1.029×10^{-2}	1.9874510068388644
Fibroblast growth factor receptor binding	GO:0005104	1.035×10^{-2}	1.985190376229314
Polysaccharide binding	GO:0030247	1.035×10^{-2}	1.985190376229314
Purine ribonucleotide binding	GO:0032555	1.069×10^{-2}	1.9710975656270313
Urokinase plasminogen activator receptor activity	GO:0030377	1.071×10^{-2}	1.970037984407178
Kisspeptin receptor binding	GO:0031773	1.071×10^{-2}	1.970037984407178
Ribonucleotide binding	GO:0032553	1.071×10^{-2}	1.970037984407178
C-8 sterol isomerase activity	GO:0000247	1.071×10^{-2}	1.970037984407178
Syntaxin-1 binding	GO:0017075	1.071×10^{-2}	1.970037984407178
Phosphoric ester hydrolase activity	GO:0042578	1.071×10^{-2}	1.970037984407178
Hydroxymethylglutaryl-CoA reductase activity	GO:0042282	1.071×10^{-2}	1.970037984407178
Purine nucleotide binding	GO:0017076	1.071×10^{-2}	1.970037984407178
Palmitoyl-CoA 9-desaturase activity	GO:0032896	1.071×10^{-2}	1.970037984407178
Laminin binding	GO:0043236	1.071×10^{-2}	1.970037984407178
fibronectin binding	GO:0001968	1.071×10^{-2}	1.970037984407178
Hydroxypyruvate isomerase activity	GO:0008903	1.071×10^{-2}	1.970037984407178
Cytokine activity	GO:0005125	1.071×10^{-2}	1.970037984407178
Dihydroceramide glucosyltransferase activity	GO:0102769	1.071×10^{-2}	1.970037984407178
Farnesyl-diphosphate farnesyltransferase activity	GO:0004310	1.071×10^{-2}	1.970037984407178
Metalloendopeptidase activity	GO:0004222	1.071×10^{-2}	1.970037984407178
Enzyme Inhibitor activity	GO:0004857	1.071×10^{-2}	1.970037984407178
Squalene synthase activity	GO:0051996	1.071×10^{-2}	1.970037984407178
Ceramide glucosyltransferase activity	GO:0008120	1.071×10^{-2}	1.970037984407178
Squalene monooxygenase activity	GO:0004506	1.071×10^{-2}	1.970037984407178
Protein tyrosine phosphatase activity	GO:0004725	1.071×10^{-2}	1.970037984407178
Hydroxymethylglutaryl-CoA reductase (NADPH) activity	GO:0004420	1.071×10^{-2}	1.970037984407178

GO, gene ontology; P_{adj} or FDR (false discovery rate), a corrected p-value.

Supplementary Table S9. Enriched GO biological process terms for up-regulated genes by OXT in HfRPE cells

Term name	Term ID	P_{adj}	-log10(P_{adj})
Cellular process	GO:0009987	5.636×10^{-40}	39.248994299366224
Biological regulation	GO:0065007	3.728×10^{-39}	38.42857151509331
Regulation of biological process	GO:0050789	1.707×10^{-35}	34.767653521538634
Response to stimulus	GO:0050896	3.598×10^{-31}	30.443973111463524
Multicellular organismal process	GO:0032501	5.435×10^{-31}	30.264794794662446
Regulation of cellular process	GO:0050794	3.278×10^{-29}	28.484395353658087
System development	GO:0048731	2.174×10^{-28}	27.662695436054065
Multicellular organism development	GO:0007275	1.012×10^{-27}	26.99499130719817
Metabolic process	GO:0008152	1.315×10^{-26}	25.88100783083141
Developmental process	GO:0032502	2.626×10^{-26}	25.580679879145805
Anatomical structure development	GO:0048856	9.588×10^{-26}	25.01828889857453
Signaling	GO:0023052	1.081×10^{-25}	24.96626082530698
Cellular response to stimulus	GO:0051716	1.081×10^{-25}	24.96626082530698
Organic substance metabolic process	GO:0071704	1.081×10^{-25}	24.96626082530698
Cell communication	GO:0007154	1.081×10^{-25}	24.96626082530698
Signal transduction	GO:0007165	3.250×10^{-24}	23.488178969634866
Positive regulation of biological process	GO:0048518	3.539×10^{-23}	22.45107705833425
Cholesterol biosynthetic process	GO:0006695	3.644×10^{-23}	22.438474911968157
Secondary alcohol biosynthetic process	GO:1902653	3.644×10^{-23}	22.438474911968157
Sterol biosynthetic process	GO:0016126	1.248×10^{-22}	21.903840594726393
Alcohol biosynthetic process	GO:0046165	1.800×10^{-21}	20.744825309864176
Positive regulation of cellular process	GO:0048522	2.452×10^{-21}	20.610401304046007
Cell surface receptor signaling pathway	GO:0007166	3.011×10^{-21}	20.52132205521476
Regulation of biological quality	GO:0065008	3.134×10^{-21}	20.503893177312552
Primary metabolic process	GO:0044238	8.999×10^{-21}	20.04579526166116
Regulation of metabolic process	GO:0019222	1.194×10^{-20}	19.923155494113104
Localization	GO:0051179	1.194×10^{-20}	19.923155494113104
Regulation of multicellular organismal process	GO:0051239	1.914×10^{-20}	19.71796061522868

Response to organic substance	GO:0010033	2.758×10^{-20}	19.55936762523311
Response to chemical	GO:0042221	3.053×10^{-20}	19.51521347751633
Cholesterol metabolic process	GO:0008203	4.723×10^{-20}	19.325791016186304
Positive regulation of response to stimulus	GO:0048584	9.248×10^{-20}	19.033931522909782
Organic hydroxy compound biosynthetic process	GO:1901617	1.174×10^{-19}	18.930422232475166
Secondary alcohol metabolic process	GO:1902652	1.219×10^{-19}	18.91383894314866
Cellular metabolic process	GO:0044237	1.886×10^{-19}	18.724427730336227
Sterol metabolic process	GO:0016125	2.293×10^{-19}	18.639533977867355
Regulation of response to stimulus	GO:0048583	4.973×10^{-19}	18.303424306790642
Regulation of cell communication	GO:0010646	7.247×10^{-19}	18.139815405786862
Regulation of signaling	GO:0023051	1.031×10^{-18}	17.9867353851213
Steroid biosynthetic process	GO:0006694	1.924×10^{-18}	17.71569718060544
Cellular response to organic substance	GO:0071310	2.784×10^{-18}	17.55528468034964
Cellular response to chemical stimulus	GO:0070887	7.229×10^{-18}	17.140926264877212
Positive regulation of metabolic process	GO:0009893	7.881×10^{-18}	17.10342608903777
Positive regulation of cell communication	GO:0010647	9.341×10^{-18}	17.02959860468892
Positive regulation of signaling	GO:0023056	9.917×10^{-18}	17.00360438629913
Animal organ development	GO:0048513	1.693×10^{-17}	16.77142208827361
Response to external stimulus	GO:0009605	1.839×10^{-17}	16.73532298368638
Response to stress	GO:0006950	4.316×10^{-17}	16.36490572042233
Alcohol metabolic process	GO:0006066	1.194×10^{-16}	15.923086366411141
Macromolecule metabolic process	GO:0043170	1.491×10^{-16}	15.826481917472732
Cellular developmental process	GO:0048869	4.023×10^{-16}	15.395465924793236
Steroid metabolic process	GO:0008202	4.215×10^{-16}	15.37520702771085
Positive regulation of signal transduction	GO:0009967	4.215×10^{-16}	15.37520702771085
Regulation of primary metabolic process	GO:0080090	5.354×10^{-16}	15.271294265068038
Anatomical structure morphogenesis	GO:0009653	1.262×10^{-15}	14.898829963537477
Regulation of signal transduction	GO:0009966	1.284×10^{-15}	14.891505550054227
Organonitrogen compound metabolic process	GO:1901564	2.672×10^{-15}	14.573094086895612
Locomotion	GO:0040011	2.798×10^{-15}	14.553151583938968
Cellular component organization	GO:0016043	3.036×10^{-15}	14.51769686476687

Lipid biosynthetic process	GO:0008610	3.409×10^{-15}	14.467358179079826
Phosphate-containing compound metabolic process	GO:0006796	3.633×10^{-15}	14.439758543848697
Phosphorus metabolic process	GO:0006793	4.717×10^{-15}	14.326303728362836
Cell differentiation	GO:0030154	5.999×10^{-15}	14.221903672632495
Organic hydroxy compound metabolic process	GO:1901615	5.999×10^{-15}	14.221903672632495
Regulation of lipid biosynthetic process	GO:0046890	7.411×10^{-15}	14.130106062957951
Cellular component organization or biogenesis	GO:0071840	9.434×10^{-15}	14.025287543010602
Nitrogen compound metabolic process	GO:0006807	1.041×10^{-14}	13.982678383814232
Biosynthetic process	GO:0009058	1.546×10^{-14}	13.810871427644898
Regulation of steroid metabolic process	GO:0019218	1.591×10^{-14}	13.79840313558002
Small molecule biosynthetic process	GO:0044283	1.664×10^{-14}	13.77875728137286
Regulation of cholesterol metabolic process	GO:0090181	2.017×10^{-14}	13.695367303186883
Regulation of steroid biosynthetic process	GO:0050810	2.632×10^{-14}	13.579695758112212
Organic substance biosynthetic process	GO:1901576	3.948×10^{-14}	13.403622154794121
Wound healing	GO:0042060	4.479×10^{-14}	13.34880515467915
Cell adhesion	GO:0007155	4.838×10^{-14}	13.3153780764793
Biological adhesion	GO:0022610	5.231×10^{-14}	13.281433850929368
Regulation of phosphate metabolic process	GO:0019220	5.231×10^{-14}	13.281433850929368
Regulation of phosphorus metabolic process	GO:0051174	5.231×10^{-14}	13.281433850929368
Secretion	GO:0046903	5.292×10^{-14}	13.276400743537067
Regulation of molecular function	GO:0065009	6.898×10^{-14}	13.161271308181187
Response to wounding	GO:0009611	9.559×10^{-14}	13.019569009479138
Positive regulation of cellular metabolic process	GO:0031325	1.107×10^{-13}	12.955689339517685
Positive regulation of macromolecule metabolic process	GO:0010604	1.200×10^{-13}	12.920766267512949
Regulation of sterol biosynthetic process	GO:0106118	1.312×10^{-13}	12.882070241507579
Regulation of cholesterol biosynthetic process	GO:0045540	1.312×10^{-13}	12.882070241507579
Regulation of catalytic activity	GO:0050790	1.312×10^{-13}	12.882070241507579
Regulation of alcohol biosynthetic process	GO:1902930	1.334×10^{-13}	12.874932257230519
Regulation of lipid metabolic process	GO:0019216	1.538×10^{-13}	12.812916621875905
Regulation of response to stress	GO:0080134	1.764×10^{-13}	12.753622989540558

Lipid metabolic process	GO:0006629	2.168×10^{-13}	12.664028904386821
Protein metabolic process	GO:0019538	2.179×10^{-13}	12.661732666356976
Regulation of developmental process	GO:0050793	2.333×10^{-13}	12.632000908070724
Establishment of localization	GO:0051234	2.894×10^{-13}	12.53853007389822
Cell activation	GO:0001775	3.702×10^{-13}	12.431594024922713
Secretion by cell	GO:0032940	4.427×10^{-13}	12.353879106544463
Transport	GO:0006810	5.019×10^{-13}	12.29937481238874
Regulation of localization	GO:0032879	5.019×10^{-13}	12.29937481238874
Enzyme linked receptor protein signaling pathway	GO:0007167	5.019×10^{-13}	12.29937481238874
Immune system process	GO:0002376	5.812×10^{-13}	12.23563833898486

GO, gene ontology; P_{adj} or FDR (false discovery rate), a corrected p-value.

Supplementary Table S10. Enriched GO cellular component terms from up-regulated genes by OXT in HfRPE cells

Cellular anatomical entity	GO:0110165	1.515×10^{-43}	42.819572188537826
Intracellular anatomical structure	GO:0005622	1.220×10^{-29}	28.91369418460477
Cytoplasm	GO:0005737	1.220×10^{-29}	28.91369418460477
Membrane	GO:0016020	5.202×10^{-29}	28.283864626213326
Extracellular region	GO:0005576	1.294×10^{-28}	27.88793902783956
Cell periphery	GO:0071944	3.294×10^{-25}	24.482286349175936
Organelle	GO:0043226	3.294×10^{-25}	24.482286349175936
Plasma membrane	GO:0005886	7.030×10^{-25}	24.153046059535388
Intrinsic component of membrane	GO:0031224	3.796×10^{-24}	23.42072612254043
Membrane-bounded organelle	GO:0043227	1.243×10^{-22}	21.905393248674198
Extracellular space	GO:0005615	8.893×10^{-22}	21.05093193503294
Integral component of membrane	GO:0016021	1.199×10^{-20}	19.921340617599647
Intracellular organelle	GO:0043229	2.871×10^{-19}	18.54203198238773
Endomembrane system	GO:0012505	3.308×10^{-18}	17.48045807250561
Intracellular membrane-bounded organelle	GO:0043231	3.999×10^{-17}	16.398102242858975
Cell junction	GO:0030054	4.576×10^{-16}	15.339549695996844
Vesicle	GO:0031982	4.165×10^{-14}	13.380363919453353
Extracellular matrix	GO:0031012	4.490×10^{-13}	12.347712903052374
Organelle membrane	GO:0031090	8.863×10^{-13}	12.052396653880603
Cell surface	GO:0009986	8.863×10^{-13}	12.052396653880603
Intrinsic component of plasma membrane	GO:0031226	3.941×10^{-12}	11.404384353001076
Integral component of plasma membrane	GO:0005887	7.528×10^{-11}	10.123297639856489
Anchoring junction	GO:0070161	2.086×10^{-10}	9.680589448525316
Cytosol	GO:0005829	4.060×10^{-10}	9.391424685362086
Bounding membrane of organelle	GO:0098588	4.071×10^{-10}	9.390247183878573
Intracellular vesicle	GO:0097708	8.780×10^{-10}	9.056488735985784
Cytoplasmic vesicle	GO:0031410	8.780×10^{-10}	9.056488735985784
Plasma membrane region	GO:0098590	1.336×10^{-9}	8.874170852158798
Organelle subcompartment	GO:0031984	1.676×10^{-9}	8.775816160868322

Secretory vesicle	GO:0099503	3.391×10^{-9}	8.469620425256482
Focal adhesion	GO:0005925	3.459×10^{-9}	8.461049568415156
Collagen-containing extracellular matrix	GO:0062023	3.942×10^{-9}	8.404241592879071
Cell-substrate junction	GO:0030055	4.036×10^{-9}	8.3940555727671
Extracellular exosome	GO:0070062	1.211×10^{-8}	7.916771079957095
Secretory granule	GO:0030141	1.558×10^{-8}	7.807520727114773
Extracellular organelle	GO:0043230	2.356×10^{-8}	7.627915850781699
Extracellular vesicle	GO:1903561	2.356×10^{-8}	7.627915850781699
Synapse	GO:0045202	2.149×10^{-7}	6.667831131603802
Endoplasmic reticulum	GO:0005783	2.792×10^{-7}	6.554015759188535
Cytoplasmic vesicle membrane	GO:0030659	3.600×10^{-7}	6.443654583078667
Vesicle membrane	GO:0012506	4.829×10^{-7}	6.31614818683838
Cell projection	GO:0042995	9.466×10^{-7}	6.023811345722025
Golgi apparatus	GO:0005794	1.721×10^{-6}	5.7641212993258835
Plasma membrane bounded cell projection	GO:0120025	2.075×10^{-6}	5.6830365321725385
Secretory granule membrane	GO:0030667	4.655×10^{-6}	5.332095204455005
External side of plasma membrane	GO:0009897	4.812×10^{-6}	5.317663170789771
Intracellular non-membrane-bounded organelle	GO:0043232	1.404×10^{-5}	4.852768866448239
Non-membrane-bounded organelle	GO:0043228	1.417×10^{-5}	4.848493561642155
Endoplasmic reticulum membrane	GO:0005789	1.448×10^{-5}	4.8392796782825425
Endoplasmic reticulum subcompartment	GO:0098827	1.490×10^{-5}	4.826706240805956
Side of membrane	GO:0098552	1.577×10^{-5}	4.802217721163005
Nuclear outer membrane-endoplasmic reticulum membrane network	GO:0042175	1.706×10^{-5}	4.768123449567282
Anchored component of membrane	GO:0031225	2.205×10^{-5}	4.656686060539318
Receptor complex	GO:0043235	2.282×10^{-5}	4.641706011487204
Nucleus	GO:0005634	3.710×10^{-5}	4.430587986287493
Organelle lumen	GO:0043233	3.839×10^{-5}	4.4157570496069445
Membrane-enclosed lumen	GO:0031974	3.839×10^{-5}	4.4157570496069445
Cell cortex	GO:0005938	5.837×10^{-5}	4.233828487048155
Cytoskeleton	GO:0005856	1.416×10^{-4}	3.8488828833308752
Basolateral plasma membrane	GO:0016323	1.494×10^{-4}	3.8256812427066538
Specific granule	GO:0042581	1.914×10^{-4}	3.7181620003952127
Presynaptic membrane	GO:0042734	1.914×10^{-4}	3.7181620003952127
Synaptic membrane	GO:0097060	2.113×10^{-4}	3.675107628338128
Somatodendritic compartment	GO:0036477	2.458×10^{-4}	3.6093512182823715
Integrin complex	GO:0008305	2.933×10^{-4}	3.5326398094089515
Perinuclear region of cytoplasm	GO:0048471	3.083×10^{-4}	3.5110789601413055
Intracellular organelle lumen	GO:0070013	3.798×10^{-4}	3.4204052707114188
Protein complex involved in cell adhesion	GO:0098636	4.358×10^{-4}	3.360678266713334
Postsynapse	GO:0098794	6.815×10^{-4}	3.1665046068516953
Synaptic vesicle	GO:0008021	7.099×10^{-4}	3.1488298339862415
Cell-cell junction	GO:0005911	8.847×10^{-4}	3.0532155571211215
Exocytic vesicle	GO:0070382	1.044×10^{-3}	2.981115792865395
Endoplasmic reticulum chaperone complex	GO:0034663	1.067×10^{-3}	2.971668481420264
Golgi apparatus subcompartment	GO:0098791	1.067×10^{-3}	2.971668481420264
Glutamatergic synapse	GO:0098978	1.095×10^{-3}	2.9605860081398006
Axon terminus	GO:0043679	1.225×10^{-3}	2.912009856517894

Presynapse	GO:0098793	1.251×10^{-3}	2.9027703033856866
Nucleolus	GO:0005730	1.521×10^{-3}	2.817742645739028
Golgi stack	GO:0005795	1.762×10^{-3}	2.7538947190871568
Neuron projection	GO:0043005	1.804×10^{-3}	2.7437482758655345
Neuron projection terminus	GO:0044306	1.877×10^{-3}	2.726489710002695
Golgi membrane	GO:0000139	1.877×10^{-3}	2.726489710002695
Vacuolar membrane	GO:0005774	2.313×10^{-3}	2.6357781831247826
Tertiary granule	GO:0070820	2.325×10^{-3}	2.633511503522435
Presynaptic active zone cytoplasmic component	GO:0098831	2.460×10^{-3}	2.6090875750097653
Endoplasmic reticulum lumen	GO:0005788	3.010×10^{-3}	2.521476223164561
Extrinsic component of plasma membrane	GO:0019897	3.053×10^{-3}	2.5152896053869145
Leading edge membrane	GO:0031256	3.053×10^{-3}	2.5152896053869145
Presynaptic active zone	GO:0048786	3.053×10^{-3}	2.5152896053869145
Extrinsic component of membrane	GO:0019898	3.137×10^{-3}	2.5034728246572864
Nucleoplasm	GO:0005654	3.265×10^{-3}	2.486058072754075
Distal axon	GO:0150034	3.294×10^{-3}	2.482260925888621
Secretory granule lumen	GO:0034774	3.351×10^{-3}	2.474797394708258
Axon	GO:0030424	3.420×10^{-3}	2.466005784765486
Cytoplasmic vesicle lumen	GO:0060205	3.468×10^{-3}	2.459980078731775
Caveola	GO:0005901	3.491×10^{-3}	2.457080540781166
Vesicle lumen	GO:0031983	3.491×10^{-3}	2.457080540781166
Nuclear lumen	GO:0031981	3.516×10^{-3}	2.453935055384948
Clathrin-coated vesicle	GO:0030136	3.705×10^{-3}	2.431242846835

GO, gene ontology; P_{adj} or FDR (false discovery rate), a corrected p-value.

Supplementary Table S11. Enriched KEGG terms from down-regulated genes by OXT in HfRPE cells

Term name	Term ID	P_{adj}	-log10(P_{adj})
Prostate cancer	KEGG:05215	3.826×10^{-3}	2.417266909954961
Tight junction	KEGG:04530	1.283×10^{-2}	1.8917596936726857
Pathogenic Escherichia coli infection	KEGG:05130	1.505×10^{-2}	1.822523978398466
Regulation of actin cytoskeleton	KEGG:04810	1.614×10^{-2}	1.7920080352569085
Calcium signaling pathway	KEGG:04020	1.774×10^{-2}	1.7509252104799957
EGFR tyrosine kinase inhibitor resistance	KEGG:01521	2.131×10^{-2}	1.6713909714973918
MAPK signaling pathway	KEGG:04010	2.131×10^{-2}	1.6713909714973918
Gastric acid secretion	KEGG:04971	2.131×10^{-2}	1.6713909714973918
Gap junction	KEGG:04540	2.296×10^{-2}	1.639066183265045
Protein digestion and absorption	KEGG:04974	2.839×10^{-2}	1.54680031659501
Serotonergic synapse	KEGG:04726	3.117×10^{-2}	1.5062457987655398

KEGG, Kyoto Encyclopedia of Genes and Genomes database; P_{adj} or FDR (false discovery rate), a corrected p-value.

Supplementary Table S12. Enriched GO molecular function terms from down-regulated genes by OXT in HfRPE cells

Term name	Term ID	P_{adj}	$-\log_{10}(P_{adj})$
Binding	GO:0005488	2.566×10^{-12}	11.590671341724613
Protein binding	GO:0005515	1.962×10^{-9}	8.707385658108226

Ion binding	GO:0043167	1.861×10^{-6}	5.730169202035372
Transporter activity	GO:0005215	5.916×10^{-6}	5.228002258015082
Passive transmembrane transporter activity	GO:0022803	1.232×10^{-5}	4.909235415114469
Channel activity	GO:0015267	1.232×10^{-5}	4.909235415114469
Metal ion binding	GO:0046872	2.463×10^{-5}	4.608462491848632
Cation binding	GO:0043169	2.594×10^{-5}	4.586075836210209
Inorganic molecular entity transmembrane transporter activity	GO:0015318	2.594×10^{-5}	4.586075836210209
Ion transmembrane transporter activity	GO:0015075	6.154×10^{-5}	4.2108226479476
Metal ion transmembrane transporter activity	GO:0046873	6.430×10^{-5}	4.191795586454621
Structural molecule activity	GO:0005198	6.430×10^{-5}	4.191795586454621
Ion channel activity	GO:0005216	6.430×10^{-5}	4.191795586454621
Transmembrane transporter activity	GO:0022857	1.060×10^{-4}	3.974755464217867
Cation channel activity	GO:0005261	2.514×10^{-4}	3.5996476814475318
Potassium ion transmembrane transporter activity	GO:0015079	2.514×10^{-4}	3.5996476814475318
Heparin binding	GO:0008201	2.515×10^{-4}	3.599466554816906
Extracellular matrix binding	GO:0050840	2.515×10^{-4}	3.599466554816906
Inorganic cation transmembrane transporter activity	GO:0022890	2.515×10^{-4}	3.599466554816906
Cation transmembrane transporter activity	GO:0008324	3.610×10^{-4}	3.4424707186681403
Calcium ion binding	GO:0005509	6.172×10^{-4}	3.2095883466761026
Antiporter activity	GO:0015297	7.613×10^{-4}	3.1184372568243215
Glycosaminoglycan binding	GO:0005539	7.613×10^{-4}	3.1184372568243215
Sulfur compound binding	GO:1901681	1.108×10^{-3}	2.955475225534366
Calcium channel activity	GO:0005262	1.900×10^{-3}	2.721254096921713
Carbohydrate derivative binding	GO:0097367	2.050×10^{-3}	2.6882749155556227
Calcium ion transmembrane transporter activity	GO:0015085	2.557×10^{-3}	2.5922191082320407
Transcription coactivator binding	GO:0001223	2.652×10^{-3}	2.576495372671437
Voltage-gated cation channel activity	GO:0022843	2.884×10^{-3}	2.539991029546977
Intramembrane lipid transporter activity	GO:0140303	3.882×10^{-3}	2.410897808823162
Monovalent inorganic cation transmembrane transporter activity	GO:0015077	3.882×10^{-3}	2.410897808823162
Divalent inorganic cation transmembrane transporter activity	GO:0072509	3.882×10^{-3}	2.410897808823162
Extracellular matrix structural constituent	GO:0005201	4.061×10^{-3}	2.3913787704699696
Nucleoside phosphate binding	GO:1901265	5.583×10^{-3}	2.253121344238904
Nucleotide binding	GO:0000166	5.583×10^{-3}	2.253121344238904
Voltage-gated ion channel activity	GO:0005244	6.006×10^{-3}	2.2214120744015844
Voltage-gated channel activity	GO:0022832	6.006×10^{-3}	2.2214120744015844
POU domain binding	GO:0070974	6.238×10^{-3}	2.2049518822448873
Ion antiporter activity involved in regulation of presynaptic membrane potential	GO:0099520	6.238×10^{-3}	2.2049518822448873
DNA-binding transcription activator activity, RNA polymerase II-specific	GO:0001228	7.503×10^{-3}	2.124776903063652
DNA-binding transcription activator activity	GO:0001216	7.542×10^{-3}	2.1225192258739995
Heterocyclic compound binding	GO:1901363	7.634×10^{-3}	2.1172224359110583

Secondary active transmembrane transporter activity	GO:0015291	8.307×10^{-3}	2.0805554957669234
Organic cyclic compound binding	GO:0097159	8.307×10^{-3}	2.0805554957669234
Anion binding	GO:0043168	8.807×10^{-3}	2.055165204199345
Transcription coregulator binding	GO:0001221	9.197×10^{-3}	2.0363569818122755
Active ion transmembrane transporter activity	GO:0022853	9.288×10^{-3}	2.0320766363566283
Proline dehydrogenase activity	GO:0004657	9.288×10^{-3}	2.0320766363566283
Integrin binding involved in cell-matrix adhesion	GO:0098640	9.288×10^{-3}	2.0320766363566283
Small molecule binding	GO:0036094	9.288×10^{-3}	2.0320766363566283
Collagen binding	GO:0005518	9.288×10^{-3}	2.0320766363566283
Voltage-gated ion channel activity involved in regulation of postsynaptic membrane potential	GO:1905030	9.288×10^{-3}	2.0320766363566283
Urea channel activity	GO:0015265	9.288×10^{-3}	2.0320766363566283
Carbohydrate binding	GO:0030246	1.050×10^{-2}	1.9790039098179584
Chromatin binding	GO:0003682	1.094×10^{-2}	1.9611604678591745
Inositol monophosphate 3-phosphatase activity	GO:0052832	1.226×10^{-2}	1.9114874300974793
Inositol monophosphate 1-phosphatase activity	GO:0008934	1.226×10^{-2}	1.9114874300974793
Inositol monophosphate phosphatase activity	GO:0052834	1.226×10^{-2}	1.9114874300974793
Flavin adenine dinucleotide binding	GO:0050660	1.226×10^{-2}	1.9114874300974793
Inositol monophosphate 4-phosphatase activity	GO:0052833	1.226×10^{-2}	1.9114874300974793
Hormone binding	GO:0042562	1.248×10^{-2}	1.903800474635782
Voltage-gated potassium channel activity	GO:0005249	1.343×10^{-2}	1.8720418426500274
Voltage-gated potassium channel activity involved in atrial cardiac muscle cell action potential repolarization	GO:0086089	1.466×10^{-2}	1.8339212106965441
G-protein activated inward rectifier potassium channel activity	GO:0015467	1.466×10^{-2}	1.8339212106965441
A-type (transient outward) potassium channel activity	GO:0005250	1.466×10^{-2}	1.8339212106965441
Structural constituent of presynaptic active zone	GO:0098882	1.466×10^{-2}	1.8339212106965441
Androgen binding	GO:0005497	1.466×10^{-2}	1.8339212106965441
Gated channel activity	GO:0022836	1.523×10^{-2}	1.8173177457309369
Active transmembrane transporter activity	GO:0022804	1.575×10^{-2}	1.8026318163435948
Protein domain specific binding	GO:0019904	1.588×10^{-2}	1.7992201183217094
Steroid binding	GO:0005496	1.588×10^{-2}	1.7992201183217094
Calcium, potassium:sodium antiporter activity	GO:0008273	1.639×10^{-2}	1.7855092901512022
Extracellular matrix protein binding	GO:1990430	1.639×10^{-2}	1.7855092901512022
N,N-dimethylaniline monooxygenase activity	GO:0004499	1.639×10^{-2}	1.7855092901512022
Fibroblast growth factor-activated receptor activity	GO:0005007	1.639×10^{-2}	1.7855092901512022
Steroid hormone binding	GO:1990239	1.915×10^{-2}	1.7178024407764152
RNA polymerase II transcription coactivator binding	GO:0001225	1.915×10^{-2}	1.7178024407764152
Potassium channel activity	GO:0005267	2.030×10^{-2}	1.6924354397769297
Protein-containing complex binding	GO:0044877	2.095×10^{-2}	1.6787760643993546
Urea transmembrane transporter activity	GO:0015204	2.124×10^{-2}	1.6727682604757277
Cell-matrix adhesion mediator activity	GO:0098634	2.124×10^{-2}	1.6727682604757277

Purine ribonucleoside triphosphate binding	GO:0035639	2.150×10^{-2}	1.6676605291301845
Transcription regulator activity	GO:0140110	2.381×10^{-2}	1.6233244316554363
RNA polymerase II transcription regulatory region sequence-specific DNA binding	GO:0000977	2.381×10^{-2}	1.6233244316554363
DNA-binding transcription factor activity, RNA polymerase II-specific	GO:0000981	2.381×10^{-2}	1.6233244316554363
Extracellular matrix constituent conferring elasticity	GO:0030023	2.381×10^{-2}	1.6233244316554363
Oxalate transmembrane transporter activity	GO:0019531	2.381×10^{-2}	1.6233244316554363
Purine ribonucleotide binding	GO:0032555	2.381×10^{-2}	1.6233244316554363
Integrin binding	GO:0005178	2.381×10^{-2}	1.6233244316554363
CD4 receptor binding	GO:0042609	2.381×10^{-2}	1.6233244316554363
MDM2/MDM4 family protein binding	GO:0097371	2.381×10^{-2}	1.6233244316554363
Purine nucleotide binding	GO:0017076	2.381×10^{-2}	1.6233244316554363
Ribonucleotide binding	GO:0032553	2.381×10^{-2}	1.6233244316554363
Anion transmembrane transporter activity	GO:0008509	2.462×10^{-2}	1.6086531924204024
Calcium:sodium antiporter activity	GO:0005432	2.513×10^{-2}	1.5997855599937763
Sodium ion transmembrane transporter activity	GO:0015081	2.513×10^{-2}	1.5997855599937763
Phospholipid scramblase activity	GO:0017128	2.513×10^{-2}	1.5997855599937763
Arrestin family protein binding	GO:1990763	2.513×10^{-2}	1.5997855599937763
DNA-binding transcription factor activity	GO:0003700	2.578×10^{-2}	1.5887388004684426

GO, gene ontology; P_{adj} or FDR (false discovery rate), a corrected p-value.

Supplementary Table S13. Enriched GO biological process terms from down-regulated genes by OXT in HfRPE cells

Term name	Term ID	P_{adj}	-log10 (P_{adj})
Cellular process	GO:0009987	2.029×10^{-13}	12.692740963917366
Localization	GO:0051179	3.789×10^{-10}	9.421469627408213
Biological regulation	GO:0065007	3.050×10^{-8}	7.5156901087564245
Response to stimulus	GO:0050896	6.891×10^{-8}	7.161738440881226
Regulation of biological process	GO:0050789	8.820×10^{-7}	6.054507407700432
Regulation of cellular process	GO:0050794	1.083×10^{-6}	5.965332792525254
Signaling	GO:0023052	1.593×10^{-6}	5.797679023051116
Cell communication	GO:0007154	1.593×10^{-6}	5.797679023051116
Cellular response to stimulus	GO:0051716	3.258×10^{-6}	5.487063626841329
Multicellular organismal process	GO:0032501	5.158×10^{-6}	5.287506475986964
Epithelial cell differentiation involved in prostate gland development	GO:0060742	2.148×10^{-5}	4.668046413383457
Developmental process	GO:0032502	2.237×10^{-5}	4.650405361169093
Lateral sprouting from an epithelium	GO:0060601	2.392×10^{-5}	4.621329302252169
Morphogenesis of an epithelial bud	GO:0060572	4.362×10^{-5}	4.360308836374909
Transport	GO:0006810	4.583×10^{-5}	4.338820207530083
Animal organ formation	GO:0048645	4.583×10^{-5}	4.338820207530083
Regulation of biological quality	GO:0065008	5.475×10^{-5}	4.261578620421884
Establishment of localization	GO:0051234	6.018×10^{-5}	4.220519552269339
Squamous basal epithelial stem cell differentiation involved in prostate gland acinus development	GO:0060529	6.737×10^{-5}	4.171505330258466

Multicellular organism development	GO:0007275	1.204×10^{-4}	3.919335523169756
Cellular response to chemical stimulus	GO:0070887	1.204×10^{-4}	3.919335523169756
Anatomical structure formation involved in morphogenesis	GO:0048646	1.204×10^{-4}	3.919335523169756
Prostate gland epithelium morphogenesis	GO:0060740	1.207×10^{-4}	3.9181660712366178
Morphogenesis of an epithelial fold	GO:0060571	1.207×10^{-4}	3.9181660712366178
Prostate gland morphogenesis	GO:0060512	1.461×10^{-4}	3.8353875263554764
Tube morphogenesis	GO:0035239	1.618×10^{-4}	3.7910933489236402
Cellular component organization	GO:0016043	1.618×10^{-4}	3.7910933489236402
Cell-cell signaling	GO:0007267	1.998×10^{-4}	3.6994061466065733
Cellular component organization or biogenesis	GO:0071840	2.257×10^{-4}	3.646378312125121
Anatomical structure development	GO:0048856	2.770×10^{-4}	3.557510055805106
Specification of animal organ identity	GO:0010092	3.037×10^{-4}	3.5175642808302214
Negative regulation of cellular metabolic process	GO:0031324	3.130×10^{-4}	3.5044050698894
Cell differentiation	GO:0030154	3.192×10^{-4}	3.4958939777555242
Cellular developmental process	GO:0048869	3.828×10^{-4}	3.417060893591739
System development	GO:0048731	3.981×10^{-4}	3.400047137198232
Regulation of localization	GO:0032879	4.109×10^{-4}	3.386243743478247
Tube development	GO:0035295	4.725×10^{-4}	3.325584853713102
Prostate gland development	GO:0030850	5.076×10^{-4}	3.2944617666896305
Morphogenesis of embryonic epithelium	GO:0016331	5.280×10^{-4}	3.277328227290312
Inorganic Ion Transmembrane Transport	GO:0098660	6.063×10^{-4}	3.2173365934184135
Urogenital system development	GO:0001655	6.830×10^{-4}	3.165553002486967
Response to chemical	GO:0042221	6.830×10^{-4}	3.165553002486967
Regulation of morphogenesis of a branching structure	GO:0060688	6.830×10^{-4}	3.165553002486967
Regionalization	GO:0003002	6.880×10^{-4}	3.1624368830456815
Ion transport	GO:0006811	7.060×10^{-4}	3.1511652868662043
Regulation of cell division	GO:0051302	7.060×10^{-4}	3.1511652868662043
Programmed cell death	GO:0012501	7.955×10^{-4}	3.099360454992931
Morphogenesis of a branching epithelium	GO:0061138	9.575×10^{-4}	3.018846728994864
Signal transduction	GO:0007165	9.817×10^{-4}	3.00803682522394
Positive regulation of cell population proliferation	GO:0008284	1.064×10^{-3}	2.973049899717762
Regulation of epithelial cell proliferation	GO:0050678	1.083×10^{-3}	2.965213011404578
Prostatic bud formation	GO:0060513	1.083×10^{-3}	2.965213011404578
Elastic fiber assembly	GO:0048251	1.083×10^{-3}	2.965213011404578
Ion transmembrane transport	GO:0034220	1.083×10^{-3}	2.965213011404578
Cranial skeletal system development	GO:1904888	1.083×10^{-3}	2.965213011404578
Morphogenesis of a branching structure	GO:0001763	1.086×10^{-3}	2.9641491286177657
Cellular response to organic substance	GO:0071310	1.091×10^{-3}	2.9623480711067907
Cell death	GO:0008219	1.140×10^{-3}	2.943212185477096
System process	GO:0003008	1.141×10^{-3}	2.942647424769794
Prostate gland growth	GO:0060736	1.152×10^{-3}	2.93857117577472
Prostate glandular acinus development	GO:0060525	1.152×10^{-3}	2.93857117577472
Negative regulation of cellular process	GO:0048523	1.152×10^{-3}	2.93857117577472
Cellular response to endogenous stimulus	GO:0071495	1.152×10^{-3}	2.93857117577472

Intracellular signal transduction	GO:0035556	1.164×10^{-3}	2.9341104487827736
Response to organic substance	GO:0010033	1.299×10^{-3}	2.8864499325752964
Potassium ion transmembrane transport	GO:0071805	1.329×10^{-3}	2.8765550324857125
Pattern specification process	GO:0007389	1.490×10^{-3}	2.826773889867346
Seminiferous tubule development	GO:0072520	1.492×10^{-3}	2.8262635056603322
Genitalia morphogenesis	GO:0035112	1.492×10^{-3}	2.8262635056603322
Negative regulation of metabolic process	GO:0009892	1.505×10^{-3}	2.8224442395262654
Cellular localization	GO:0051641	1.579×10^{-3}	2.8015020951343077
Epithelial cell proliferation	GO:0050673	1.600×10^{-3}	2.7959488967786035
Apoptotic process	GO:0006915	1.774×10^{-3}	2.7511143820715493
Regulation of signaling	GO:0023051	1.798×10^{-3}	2.745213475538878
Inorganic cation transmembrane transport	GO:0098662	1.798×10^{-3}	2.745213475538878
Potassium ion transport	GO:0006813	1.798×10^{-3}	2.745213475538878
Blood vessel development	GO:0001568	1.984×10^{-3}	2.7024571495993657
Otic vesicle development	GO:0071599	2.027×10^{-3}	2.693066575209626
Epithelial cell differentiation	GO:0030855	2.027×10^{-3}	2.693066575209626
Transmembrane transport	GO:0055085	2.275×10^{-3}	2.6429770472428764
Cell fate commitment	GO:0045165	2.322×10^{-3}	2.634166856049344
Vasculature development	GO:0001944	2.322×10^{-3}	2.634166856049344
Animal organ development	GO:0048513	2.322×10^{-3}	2.634166856049344
Circulatory system development	GO:0072359	2.322×10^{-3}	2.634166856049344
Cell migration	GO:0016477	2.551×10^{-3}	2.5932968284905797
Tissue Development	GO:0009888	2.551×10^{-3}	2.5932968284905797
Hematopoietic stem cell homeostasis	GO:0061484	2.593×10^{-3}	2.586264792353666
Response to endogenous stimulus	GO:0009719	2.639×10^{-3}	2.578571382286883
Branch elongation of an epithelium	GO:0060602	2.784×10^{-3}	2.5553189951766417
Synaptic vesicle priming	GO:0016082	2.784×10^{-3}	2.5553189951766417
Metabolic process	GO:0008152	2.802×10^{-3}	2.55255594358548
Negative regulation of biological process	GO:0048519	2.802×10^{-3}	2.55255594358548
Blood circulation	GO:0008015	2.833×10^{-3}	2.547762280994588
Positive regulation of cellular process	GO:0048522	2.904×10^{-3}	2.537020949786287
Cation transmembrane transport	GO:0098655	3.035×10^{-3}	2.517848535396592
Regulation of cell death	GO:0010941	3.035×10^{-3}	2.517848535396592
Metal ion transport	GO:0030001	3.318×10^{-3}	2.47915839131816
Regulation of cell population proliferation	GO:0042127	3.432×10^{-3}	2.4644399020943166
Response to lipid	GO:0033993	3.432×10^{-3}	2.464

GO, gene ontology; P_{adj} or FDR (false discovery rate), a corrected p-value.

Supplementary Table S14. Enriched GO cellular component terms from down-regulated genes by OXT in HfRPE cells

Term name	Term ID	P_{adj}	-log10(P_{adj})
Cellular anatomical entity	GO:0110165	6.633×10^{-17}	16.178318914163697
Integral component of membrane	GO:0016021	3.682×10^{-14}	13.433948231296466
Intrinsic component of membrane	GO:0031224	5.611×10^{-14}	13.250923220785452
Membrane	GO:0016020	1.528×10^{-13}	12.815934162281662
Plasma membrane	GO:0005886	1.239×10^{-10}	9.906948349899338
Cell periphery	GO:0071944	1.706×10^{-10}	9.76793023730715
Organelle	GO:0043226	7.271×10^{-10}	9.138422052525831

Intrinsic component of plasma membrane	GO:0031226	9.511×10^{-8}	7.021762554878594
Cytoplasm	GO:0005737	2.187×10^{-7}	6.660086184039969
Integral component of plasma membrane	GO:0005887	4.761×10^{-7}	6.322337578536363
Vesicle	GO:0031982	1.228×10^{-6}	5.910642647228262
Intracellular anatomical structure	GO:0005622	1.591×10^{-6}	5.798247416596149
Membrane-bounded organelle	GO:0043227	1.762×10^{-5}	4.754015462000814
Cytoplasmic vesicle	GO:0031410	1.464×10^{-4}	3.8343259384736417
Endomembrane system	GO:0012505	1.464×10^{-4}	3.8343259384736417
Intracellular vesicle	GO:0097708	1.464×10^{-4}	3.8343259384736417
Extracellular matrix	GO:0031012	1.583×10^{-4}	3.80048642046554
Organelle membrane	GO:0031090	1.851×10^{-4}	3.7327074125133506
Plasma membrane region	GO:0098590	1.851×10^{-4}	3.7327074125133506
Excitatory synapse	GO:0060076	1.851×10^{-4}	3.7327074125133506
Cell junction	GO:0030054	2.026×10^{-4}	3.6933299902954584
Intracellular organelle	GO:0043229	2.753×10^{-4}	3.560225163038242
Synaptic membrane	GO:0097060	2.855×10^{-4}	3.5443425758353158
Extracellular organelle	GO:0043230	2.855×10^{-4}	3.5443425758353158
Extracellular vesicle	GO:1903561	2.855×10^{-4}	3.5443425758353158
Synapse	GO:0045202	2.855×10^{-4}	3.5443425758353158
Collagen-containing extracellular matrix	GO:0062023	3.490×10^{-4}	3.4571911684217698
Extracellular region	GO:0005576	3.617×10^{-4}	3.4415944458088643
Extracellular space	GO:0005615	5.734×10^{-4}	3.2415441624470644
Parallel fiber to Purkinje cell synapse	GO:0098688	8.050×10^{-4}	3.0941951409693274
Extracellular exosome	GO:0070062	1.010×10^{-3}	2.9955233864806647
Transmembrane transporter complex	GO:1902495	1.603×10^{-3}	2.795158523425778
Transporter complex	GO:1990351	1.763×10^{-3}	2.753761127717098
Neuronal cell body membrane	GO:0032809	2.114×10^{-3}	2.674949398119247
Protein-containing complex	GO:0032991	2.154×10^{-3}	2.666824484339111
Glutamatergic synapse	GO:0098978	2.325×10^{-3}	2.633631364421265
Cell body membrane	GO:0044298	2.374×10^{-3}	2.6244677187634733
Presynaptic membrane	GO:0042734	2.423×10^{-3}	2.615632968985932
Integral component of synaptic membrane	GO:0099699	2.479×10^{-3}	2.605685467748823
Plasma membrane protein complex	GO:0098797	2.479×10^{-3}	2.605685467748823
Intrinsic component of synaptic membrane	GO:0099240	3.080×10^{-3}	2.511405459244796
EMILIN complex	GO:1990971	4.722×10^{-3}	2.325841778893487
Protein complex involved in cell-matrix adhesion	GO:0098637	4.722×10^{-3}	2.325841778893487
Cytoskeleton	GO:0005856	5.397×10^{-3}	2.2678787114778376
Cation channel complex	GO:0034703	6.013×10^{-3}	2.220942466437225
Myosin complex	GO:0016459	6.088×10^{-3}	2.2155454562677623
Neuron projection	GO:0043005	7.883×10^{-3}	2.1033237371541493
Endoplasmic reticulum	GO:0005783	9.322×10^{-3}	2.030495246190832
Postsynaptic membrane	GO:0045211	1.062×10^{-2}	1.9736727517435635
Integral component of postsynaptic specialization membrane	GO:0099060	1.096×10^{-2}	1.960164532784744
GABA-ergic synapse	GO:0098982	1.102×10^{-2}	1.9579918014657907
Integrin alpha4-beta1 complex	GO:0034668	1.115×10^{-2}	1.9526239189683925
Intrinsic component of postsynaptic specialization membrane	GO:0098948	1.115×10^{-2}	1.9526239189683925

Ion channel complex	GO:0034702	1.115×10^{-2}	1.9526239189683925
Glial limiting end-foot	GO:0097451	1.115×10^{-2}	1.9526239189683925
Endoplasmic reticulum lumen	GO:0005788	1.116×10^{-2}	1.9523209600457108
Cell cortex	GO:0005938	1.174×10^{-2}	1.9304725682554735
Organelle lumen	GO:0043233	1.174×10^{-2}	1.9304725682554735
Voltage-gated potassium channel complex	GO:0008076	1.174×10^{-2}	1.9304725682554735
Axon	GO:0030424	1.174×10^{-2}	1.9304725682554735
Membrane-enclosed lumen	GO:0031974	1.174×10^{-2}	1.9304725682554735
Bounding membrane of organelle	GO:0098588	1.194×10^{-2}	1.922909846974657
Collagen trimer	GO:0005581	1.223×10^{-2}	1.9124187436153208
Photoreceptor inner segment membrane	GO:0060342	1.277×10^{-2}	1.8938228661235381
Potassium channel complex	GO:0034705	1.311×10^{-2}	1.8822577865880155
Melanosome	GO:0042470	1.380×10^{-2}	1.8600778646911527
Plasma membrane bounded cell projection	GO:0120025	1.380×10^{-2}	1.8600778646911527
Pigment granule	GO:0048770	1.380×10^{-2}	1.8600778646911527
Neuron to neuron synapse	GO:0098984	1.577×10^{-2}	1.8021117381507983
Intracellular non-membrane-bounded organelle	GO:0043232	1.638×10^{-2}	1.7856363524331225
Cell projection	GO:0042995	1.638×10^{-2}	1.7856363524331225
Non-membrane-bounded organelle	GO:0043228	1.638×10^{-2}	1.7856363524331225
Cytoplasmic vesicle membrane	GO:0030659	1.770×10^{-2}	1.751932941268313
Integral component of postsynaptic membrane	GO:0099055	1.781×10^{-2}	1.749366823368563
Postsynaptic specialization membrane	GO:0099634	1.785×10^{-2}	1.7484216167797098
Vesicle membrane	GO:0012506	1.872×10^{-2}	1.7276150843194438
Intrinsic component of postsynaptic membrane	GO:0098936	1.875×10^{-2}	1.727024923842619
External side of plasma membrane	GO:0009897	1.924×10^{-2}	1.7158236531643538
Membrane protein complex	GO:0098796	2.005×10^{-2}	1.6978845260852014
Astrocyte end-foot	GO:0097450	2.046×10^{-2}	1.6891380244923226
Intracellular organelle lumen	GO:0070013	2.054×10^{-2}	1.6873615844986976
Secretory granule	GO:0030141	2.094×10^{-2}	1.679085205401438
Intracellular membrane-bounded organelle	GO:0043231	2.324×10^{-2}	1.633762103133748
Cell surface	GO:0009986	2.646×10^{-2}	1.5774445514638604
Neurofilament	GO:0005883	2.646×10^{-2}	1.5774285979376148
Sodium:potassium-exchanging atpase complex	GO:0005890	2.852×10^{-2}	1.54485406490759
Receptor complex	GO:0043235	3.049×10^{-2}	1.5158372303841654
Platelet dense granule lumen	GO:0031089	3.250×10^{-2}	1.4881644023670602
Presynapse	GO:0098793	3.331×10^{-2}	1.4774819142121358
Supramolecular fiber	GO:0099512	3.331×10^{-2}	1.4774819142121358
Muscle myosin complex	GO:0005859	3.331×10^{-2}	1.4774819142121358
Supramolecular polymer	GO:0099081	3.331×10^{-2}	1.4774819142121358
Secretory vesicle	GO:0099503	3.449×10^{-2}	1.462292987183275
Presynaptic active zone cytoplasmic component	GO:0098831	3.476×10^{-2}	1.4588923205483098
Cation-transporting atpase complex	GO:0090533	4.080×10^{-2}	1.3893855299723292
Myosin II complex	GO:0016460	4.205×10^{-2}	1.376256991284904
Astrocyte projection	GO:0097449	4.205×10^{-2}	1.376256991284904
Platelet dense granule	GO:0042827	4.368×10^{-2}	1.3596697978456322

Side of membrane	GO:0098552	4.494×10^{-2}	1.3473376982199676

GO, gene ontology; P_{adj} or FDR (false discovery rate), a corrected p-value.

Supplementary Table S15. Top 50 differentially expressed genes (DEGs) in HfRPE cells treated with OXT

Ensembl ID	Gene Symbol	logFC	FDR
ENSG00000203721	LINC00862	1.38E+00	7.66E-16
ENSG00000127329	PTPRB	1.07E+00	2.54E-12
ENSG00000113070	HBEGF	1.15E+00	8.18E-11
ENSG00000168685	IL7R	1.68E+00	8.21E-11
ENSG00000052795	FNIP2	7.03E-01	1.63E-10
ENSG00000164171	ITGA2	1.13E+00	2.39E-10
ENSG00000131016	AKAP12	9.91E-01	4.76E-10
ENSG00000148677	ANKRD1	1.43E+00	1.18E-09
ENSG00000163331	DAPL1	-1.07E+00	2.72E-09
ENSG00000186480	INSIG1	9.03E-01	2.01E-08
ENSG00000112414	ADGRG6	6.43E-01	3.20E-08
ENSG00000112972	HMGCS1	8.74E-01	1.16E-07
ENSG00000117525	F3	8.28E-01	1.16E-07
ENSG00000138675	FGF5	7.91E-01	1.16E-07
ENSG00000145721	LIX1	-9.47E-01	1.16E-07
ENSG00000134516	DOCK2	7.90E-01	1.23E-07
ENSG00000198959	TGM2	1.28E+00	1.23E-07
ENSG00000118523	CTGF	8.61E-01	1.29E-07
ENSG00000163661	PTX3	9.33E-01	1.32E-07
ENSG00000145777	TSLP	8.44E-01	1.72E-07
ENSG00000233117	LINC00702	1.10E+00	6.34E-07
ENSG00000113578	FGF1	7.56E-01	1.13E-06
ENSG00000173166	RAPH1	8.01E-01	1.39E-06
ENSG00000154678	PDE1C	9.31E-01	1.46E-06
ENSG00000130600	H19	-7.41E-01	1.87E-06
ENSG00000132196	HSD17B7	8.37E-01	1.87E-06
ENSG00000147606	SLC26A7	-1.15E+00	1.87E-06
ENSG00000144063	MALL	1.24E+00	2.02E-06
ENSG00000110427	KIAA1549L	1.10E+00	2.07E-06
ENSG00000079459	FDFT1	6.93E-01	2.09E-06
ENSG00000079841	RIMS1	8.47E-01	2.41E-06
ENSG00000081181	ARG2	1.16E+00	2.41E-06
ENSG00000115339	GALNT3	7.50E-01	2.64E-06
ENSG00000079931	MOXD1	6.07E-01	5.05E-06

ENSG00000166923	GREM1	7.50E-01	5.05E-06
ENSG00000147155	EBP	6.67E-01	5.06E-06
ENSG00000147573	TRIM55	1.02E+00	5.81E-06
ENSG00000104549	SQLE	6.65E-01	6.23E-06
ENSG00000164741	DLC1	6.46E-01	6.52E-06
ENSG00000170962	PDGFD	-7.16E-01	6.71E-06
ENSG00000113161	HMGCR	6.42E-01	7.24E-06
ENSG00000052802	MSMO1	8.16E-01	7.27E-06
ENSG00000139211	AMIGO2	6.41E-01	9.14E-06
ENSG00000154127	UBASH3B	7.83E-01	9.15E-06
ENSG00000130164	LDLR	8.43E-01	9.94E-06
ENSG00000127824	TUBA4A	7.52E-01	1.31E-05
ENSG00000066468	FGFR2	-7.01E-01	1.45E-05
ENSG00000176697	BDNF	7.85E-01	1.45E-05
ENSG00000120437	ACAT2	8.91E-01	1.57E-05
ENSG00000073008	PVR	7.03E-01	1.73E-05

log_2FC, log_2(fold change); FDR, false discovery rate that is a corrected p-value by the Benjamini-Hochberg procedure; Fold change data without a minus sign denote up-regulation; Fold change data with a minus sign denote down-regulation.
